# Self-Reported Health and Sarcopenia Phenotypes: Data From the National Health and Aging Trends Survey

**DOI:** 10.1093/geroni/igaa057.577

**Published:** 2020-12-16

**Authors:** John Batsis, Cameron Dowd-Sivigny, Christian Haudenschild, Rebecca Crow, Tyler Gooding, Matthew Lohman, Elizabeth Vasquez

**Affiliations:** 1 Dartmouth College, Lebanon, New Hampshire, United States; 2 University at Albany: School of Public Health, Rensselaer, New York, United States; 3 Dartmouth College Geisel School of Medicine, Lebanon, United States; 4 dartmouth hitchcock, lebanon, New Hampshire, United States; 5 Dartmouth Hitchcock Medical Center, Lebanon, New Hampshire, United States; 6 University of South Carolina, Columbia, South Carolina, United States; 7 University at Albany (SUNY), Rensselaer, New York, United States

## Abstract

Background: Obesity in combination with sarcopenia (age-related loss of muscle mass, strength or function) is increasing in adults aged ≥65 years which places individuals at risk for functional decline and worse health. We ascertained the relationship between sarcopenic obesity and self-reported health in a representative US population. Methods: We identified participants ≥65 years with grip strength and body mass index (BMI) measures from the baseline wave of the National Health and Aging Trends Survey. Sarcopenia was defined using the Sarcopenia Definitions and Outcomes Consortium grip strength cut-points (males<35.5kg; females<20kg), and obesity was defined using standard World Health Organization BMI categories. We also assessed grip divided by BMI cut-points (males<1.05; females<0.79). Self-reported health was evaluated using a one-item question (Excellent/Very Good/Good vs. Fair/Poor). Logistic regression models adjusted for age, sex, smoking, education, comorbidities, and an ability to walk. Results: Of the 8,245 participants (59.7% female), median age category was 75-79, and mean grip strength and BMI were 26.0 kg and 27.0 kg/m2, respectively. Prevalence of sarcopenia and sarcopenic obesity was 12.7% and 41.1%. Compared to those without sarcopenia or obesity, the odds of impaired self-reported health using grip strength defined sarcopenia cut-points was higher in sarcopenic obesity (OR 1.87 [1.50,2.32]), sarcopenia (OR 1.79 [1.50,2.14]), and obesity (OR 1.24 [0.99,1.56]). Using the Grip divided by BMI cutpoints, we found the odds of low self-reported health in sarcopenia was OR 1.38 [1.12, 1.59]. Conclusions: Both sarcopenia and sarcopenic obesity are associated with an increased odds of decreased self-reported health.

